# Trajectories of depression in sepsis survivors: an observational cohort study

**DOI:** 10.1186/s13054-021-03577-7

**Published:** 2021-04-29

**Authors:** Monique Boede, Jochen S. Gensichen, James C. Jackson, Fiene Eißler, Thomas Lehmann, Sven Schulz, Juliana J. Petersen, Florian P. Wolf, Tobias Dreischulte, Konrad F. R. Schmidt

**Affiliations:** 1 Institute of General Practice and Family Medicine, Jena University Hospital, Bachstraße 18, 07743 Jena, Germany; 2 Institute of General Practice and Family Medicine, University Hospital of Ludwig-Maximilians-University Munich, Pettenkoferstr. 10, 80336 Munich, Germany; 3 Center for Sepsis Control and Care, Jena University Hospital, Am Klinikum 1, 07747 Jena, Germany; 4 Department of Medicine, Pulmonary and Critical Care, Critical Illness, Brain Dysfunction and Survivorship (CIBS) Center, Vanderbilt University, Suite 450, 4th Floor 2525 West End Avenue, Nashville, TN 37203 USA; 5 Paediatrics and Adolescent Medicine, Sanaklinikum Lichtenberg, Fanningerstraße 32, 10365 Berlin, Germany; 6 Center for Clinical Studies, Jena University Hospital, Salvador-Allende-Platz 27, 07747 Jena, Germany; 7 Institute of General Practice, Goethe-University Frankfurt am Main, Theodor-Stern-Kai 7, 60590 Frankfurt a. Main, Germany; 8 Institute of General Practice, Charité University Medicine, Charitéplatz 1, 10117 Berlin, Germany

**Keywords:** Sepsis, Post-intensive care syndrome (PICS), Depression, Health-related quality of life (HRQOL), Chronic pain, Posttraumatic stress disorder (PTSD), Comorbidity, Rehabilitation

## Abstract

**Background:**

Advances in critical care medicine have led to a growing number of critical illness survivors. A considerable part of them suffers from long-term sequelae, also known as post-intensive care syndrome. Among these, depressive symptoms are frequently observed. Depressive symptom trajectories and associated factors of critical illness survivors have rarely been investigated. Study objective was to explore and compare different trajectories of depressive symptoms in sepsis survivors over 1 year after discharge from ICU.

**Methods:**

Data of a randomized controlled trial on long-term post-sepsis care were analyzed post hoc. Depressive symptoms were collected at 1, 6 and 12 months post-ICU discharge using the Major Depression Inventory (MDI), among others. Statistical analyses comprised descriptive analysis, univariate and multivariate, linear and logistic regression models and Growth Mixture Modeling.

**Results:**

A total of 224 patients were included into this analysis. We identified three latent classes of depressive symptom trajectories: Over the course of 1 year, 152 patients recovered from mild symptoms, 27 patients showed severe persistent symptoms, and 45 patients recovered from severe symptoms. MDI sum scores significantly differed between the three classes of depressive symptom trajectories at 1 and 6 months after ICU discharge (*p* < 0.024 and *p* < 0.001, respectively). Compared with other classes, patients with the mild recovered trajectory showed lower levels of chronic pain (median sum score of 43.3 vs. 60.0/53.3 on the Graded Chronic Pain Scale, *p* < 0.010) and posttraumatic stress (4.6% with a sum score of ≥ 35 on the Posttraumatic Stress Scale 10 vs. 48.1%/33.3%, *p* < 0.003); and higher levels of health-related quality of life (HRQOL) using the Short Form-36 scale within 1 month after ICU discharge (*p* < 0.035).

**Conclusions:**

In the first year after discharge from ICU, sepsis survivors showed three different trajectories of depressive symptoms. Course and severity of depressive symptoms were associated with chronic pain, posttraumatic stress and reduced HRQOL at discharge from ICU. Regular screening of sepsis survivors on symptoms of depression, chronic pain and posttraumatic stress within 1 year after ICU may be considered.

*Trial registration* ISRCTN, ISRCTN 61744782. Registered April 19, 2011—Retrospectively registered, http://www.isrctn.com/ISRCTN61744782.

## Background

Sepsis is one of the most frequent critical illnesses treated on intensive care units (ICU) [1, 2]. Fortunately, advances in critical care medicine have led to a growing number of survivors. Patients who survive critical illnesses may develop a range of impairments affecting cognitive, mental and physical functions, known as post-intensive care syndrome (PICS) [3]. As a result the health-related quality of life (HRQOL) may be reduced for months and years [4, 5].

The main psychiatric impairments after sepsis and critical care are posttraumatic stress disorder (PTSD), anxiety disorder and depression [6]. These conditions are frequently coincident, aggravate each other and reduce mental HRQOL [7]. Several symptoms might overlap: For instance, patients with major depression report similar symptoms, such as numbing and dysphoria, compared to patients with PTSD [8]. Moreover, both conditions can be associated with pain [9–11]. The diagnosis of PTSD also includes symptoms of anxiety but is typically characterized by flashbacks. PTSD can be typically triggered by a major life event, whereas anxiety disorders usually develop gradually over many years. Among others, experience of delirium and life-threatening events as well as feelings of loss of control, preexisting psychopathologies and the use of sedatives are discussed as risk factors [12].

According to a systematic review, depressive symptoms can occur and persist up to 1 year in around 30% of critical illness survivors [13]. Moreover, symptoms of depression are associated with increased mortality for 2 years following ICU discharge, as shown by a large cohort study in the UK [7].

Depression is already known to follow distinct trajectories: The persistent depressive disorder must be distinguished from non-chronic major depression, which clearly differ in their course and therapeutic consequences [14]. However, little is known on symptom trajectories and associated factors of depressive symptoms following critical illness. This knowledge could help to adapt screening sequences, improve timely diagnosis and optimize therapy strategies.

In addition, major intervention studies (SMOOTH, RAPIT) failed to show effective treatment in ICU follow-up care on depression [15, 16]. Thus, we need to understand more about characteristics and symptom trajectories of this vulnerable group of patients with post-ICU depression. Consequently, the aim of this analysis was to explore different trajectories of depressive symptoms in sepsis survivors over 1 year and to assess the associations between sociodemographic and clinical data at ICU discharge and symptom trajectories accordingly.

## Methods

### Study design

Data were derived from the SMOOTH trial (*Sepsis survivors Monitoring and Coordination in Outpatient Healthcare)*, a randomized, multicenter intervention study on a primary care-based follow-up of sepsis survivors [trial registration number: ISRCTN 61744782]. The trial was conducted between February 2011 and December 2015. The study was approved by the Institutional Review Board of Jena University (No. 3001/111). Details on the study design, baseline descriptions and results have been reported elsewhere [16–18].

In brief, the intervention included training for the primary care physician (PCP) and patients as well as case management provided by trained nurses and clinical decision support for PCPs by a consulting physician. All patients and PCPs signed written informed consent. The primary outcome was mental health–related quality of life, which remained unaffected by the intervention. Data were obtained in telephone or face-to-face interviews at 1, 6 and 12 months after ICU discharge for the entire study population. Within the intervention group, additional assessments were performed at 2, 3, 4, 5 and 9 months after ICU discharge. Depressive symptoms were among the secondary outcomes, using the Major Depression Inventory (MDI) for the entire study population and the Patient Health Questionnaire 9 (PHQ-9) for the additional assessments within the intervention group.

### Applied measures

The MDI is a self-report mood questionnaire developed by the World Health Organization, with a sum score ranging from 0 to 50 [19]. The PHQ-9 is a 9-item questionnaire orientated on the Diagnostic and Statistical Manual of Mental Disorders (DSM-IV) criteria for diagnosis of depression and widely used in primary care with a total sum score that ranges from 0 to 27 [20, 21]. Both measures allow categorization into mild, moderate and severe depression, and high scores indicate more severe depression.

Health-related quality of life (HRQOL) was measured using the 36-item Short Form Survey (SF-36) [22], consisting of eight physical functioning and mental functioning subscales. Posttraumatic stress was assessed by use of the Posttraumatic Stress Scale (PTSS-10) [23] and chronic pain by the Graded Chronic Pain Scale (GCPS) [24]. Clinical data were obtained from patient records at ICU and at the primary care provider.

### Inclusion criteria

Assuming that there was no effect on the depression trajectory by the SMOOTH intervention—as suggested by the primary analysis [16]—we included intervention and control recipients with at least two completed MDI follow-ups. Patients of the intervention group, who additionally completed at least four PHQ-9 follow-ups, were included in a subgroup analysis comparing the trajectories defined by MDI and PHQ-9 with different frequencies of evaluation. These data have not yet been published.

### Statistical analysis

Growth Mixture Modeling (GMM) was applied using the R package “lcmm” [25] in R 4.0.1 [26] to define latent classes of depressive symptom severity and trajectories after sepsis based on the MDI and PHQ-9 sum scores. This advanced tool allows analyzing longitudinal data by estimating possible associated factors of the variation of the growth factors and the mean growth curve for each class. It captures individual variation around these growth curves by the factor variances for each class. [27]. The maximal likelihood was used as estimation criteria for heterogeneity. The Bayesian information criterion (BIC) [28] was used to identify the model (and number of trajectories) that fits best for our data and to medical plausibility. Graphs were created using the R package ggplot2 [29] and Microsoft Excel [30].

To describe the sample and the patients of the previously defined classes, absolute and relative frequencies were reported for categorical variables. Chi-square test was performed to test for significant differences between the classes. For data analysis of continuous variables, medians and interquartile ranges were calculated and Kruskal–Wallis test [31] was used to compare the previously defined classes. The significance level was set at *α* = 0.05. Multivariate linear and logistic regression models were applied for the outcome variables including all covariates that were found to be significant in univariate analyses. All of these statistical analyses were performed using IBM Statistical Package for Social Sciences (SPSS) Statistics for Windows, version 26 [32].

## Results

### Study population

**Fig. 1:**
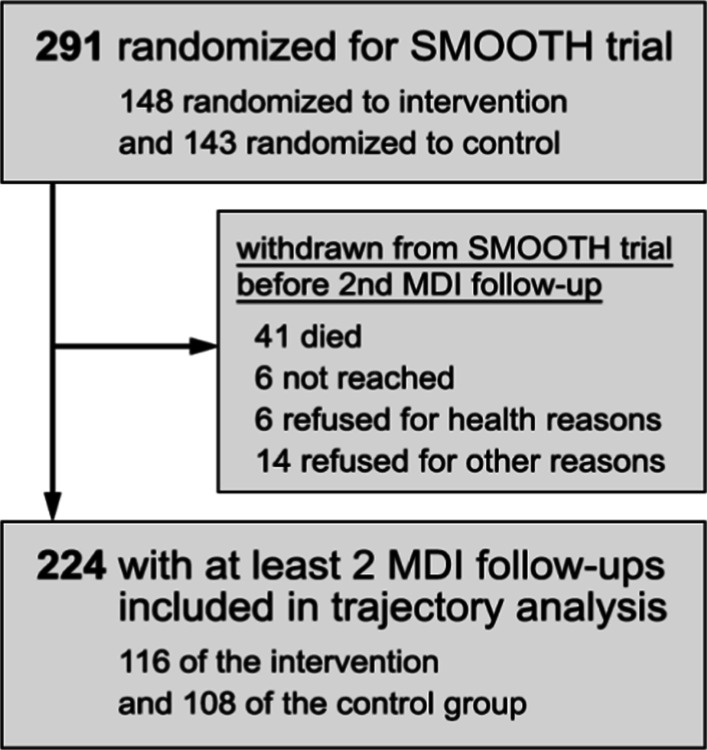
Flowchart diagram of patient inclusion

Of 291 patients included in the RCT (SMOOTH), 224 patients (77.0%) were included in this analysis; a total of 67 patients had to be excluded due to missing MDI data—all beside one because of death or other reasons for drop out, corresponding to the primary analysis [16]. Reasons for exclusion are shown in Fig. 1. The mean age of included patients was 64 years, and 68.9% were males. Both values ranged slightly above the entire study population.

Patients of the intervention group were evenly distributed between the three classes with 59.9% (*n* = 91), 51.9% (*n* = 14) and 51.1% (*n* = 23) in the mild recovered, severe persistent and severe recovered trajectory class, respectively. As we found no statistically significant association of the intervention, the number of monitoring contacts and the trajectories (Chi-square test, *p* = 0.487 and *p* = 0.766, respectively), patients from both groups, intervention and control, were included in our main analysis.

### Trajectories of depressive symptoms

**Fig. 2:**
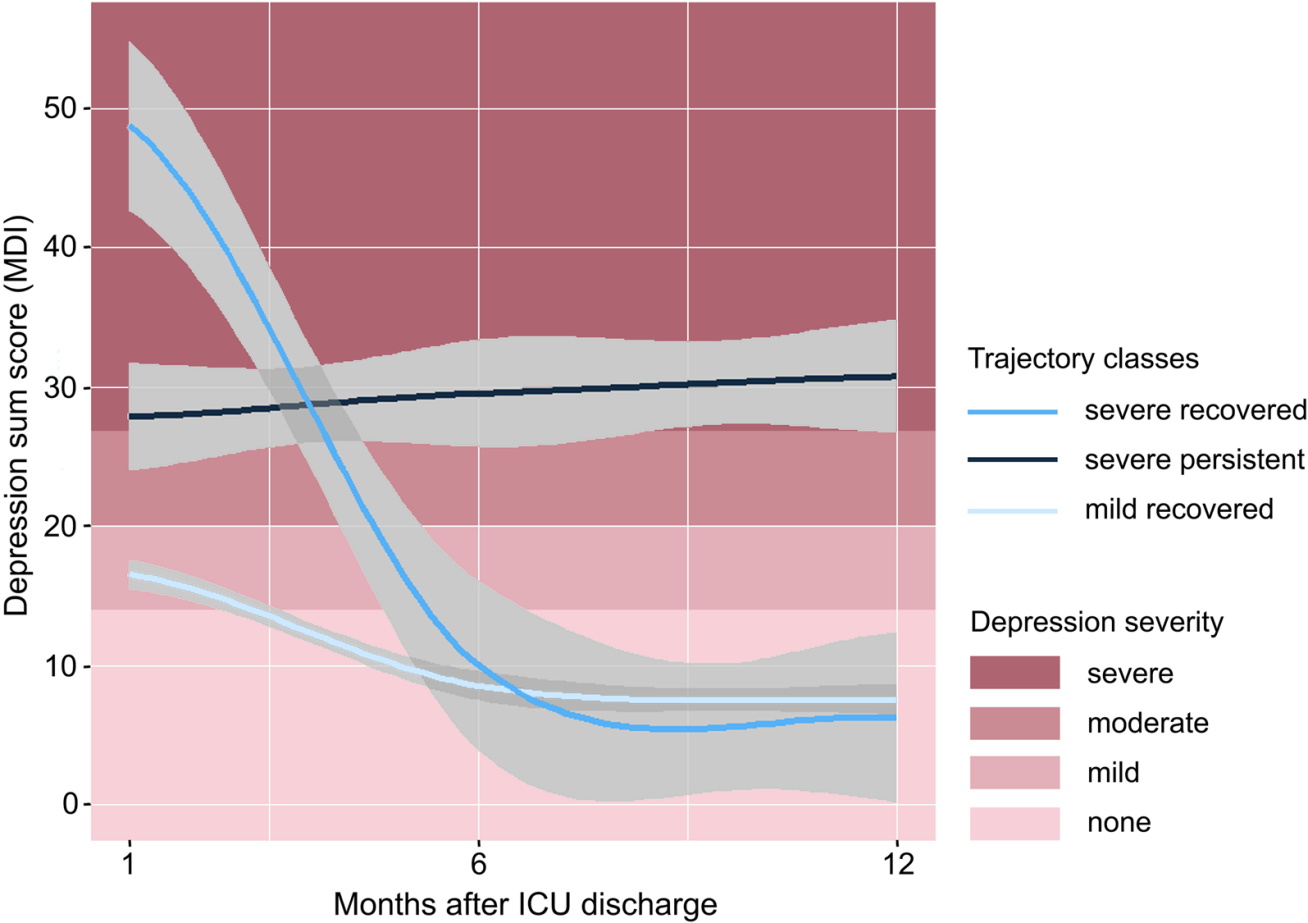
Trajectories of depressive symptoms in sepsis survivors—results of the Growth Mixture Modeling: depressive symptoms measured by Major Depression Inventory (MDI), sum scores range from 0 to 50 (high scores indicate more severe symptoms). Diagnostic thresholds for depression are labeled according to ICD-10 and DSM-IV. Three trajectories were identified: mild recovered (*n* = 152), severe persistent (*n* = 27) and severe recovered trajectory (*n* = 45)

Applying the Growth Mixture Modeling (GMM), a model with three classes of trajectories fitted best as it showed the lowest Bayesian information criteria (BIC = 823.72). Trajectory classes are shown in Fig. 2: One class (*n* = 152) showed mild depressive symptoms 1 month after ICU discharge and symptoms recovered within 6 months (mild recovered trajectory [mr]). Another class (*n* = 27) initially suffered from moderate to severe depressive symptoms that persisted over the period of 1 year (severe persistent trajectory [sp]). The third class (*n* = 45) presented complete recovery after 6 months although they had severe depressive symptoms 1 month after ICU discharge (severe recovered trajectory [sr]).

### Patient characteristics of trajectory classes

No statistically significant differences between patients in the different trajectory classes were found at ICU discharge, neither related to age, gender, clinical conditions, nor to intensive care parameters, as shown in Table 1. However, the severe persistent trajectory class showed further differences from the other classes, which were not statistically significant—possibly due to the small number of patients in this class (*n* = 27): Fewer patients were employed and had university degrees. Before sepsis, more patients had suffered from a psychiatric diagnosis and use of antidepressants and analgesics was observed more frequently. Length of ICU stay was shorter, and less sedatives were given.

**Table 1:** Characteristics of trajectory classes 1 month after discharge from ICU

	Mild recovered (*n* = 152)	Severe persistent (*n* = 27)	Severe recovery (*n* = 45)	Total (*n* = 224)	Missings no. (%)	*p* value
*Sociodemographics*						
Age, years mean (SD)	61.4 (14.3)	58.8 (13.7)	59.4 (12.2)	64.0 (14.4)	0 (0.0)	0.367^a^
Women, no. (%)	47 (30.9)	11 (40.7)	12 (26.7)	70 (31.3)	0 (0.0)	0.454^b^
Married, no. (%)	82 (53.9)	18 (66.6)	21 (46.7)	121 (54.0)	4 (1.8)	0.184^b^
Employed, no. (%)	45 (30.2)	6 (22.2)	13 (28.9)	64 (29.0)	3 (1.3)	0.702^b^
*Educational status*						
< High school, no. (%)	52 (34.2)	7 (25.9)	13 (28.9)	72 (32.1)	1 (0.4)	0.782^b^
University degree, no. (%)	33 (14.7)	2 (0.9)	9 (4.0)	44 (19.7)	1 (0.4)	0.744^b^
*Comorbidity measures*						
Charlson comorbidity index, median (IQR)	3.0 (3.0)	3.0 (4.0)	5.0 (5.0)	3.0 (4.0)	1 (0.4)	0.085^a^
Active smoking, no. (%)	18 (12.9)	6 (23.1)	7 (17.1)	31 (15.0)	17 (7.6)	0.640^b^
Body mass index, median (IQR)	26.9 (6.4)	28.3 (6.5)	25.7 (8.0)	27.1 (6.7)	6 (2.7)	0.137^a^
Psychiatric diagnosis before sepsis^c^, no. of patients (%)	18 (11.8)	9 (33.3)	7 (15.6)	34 (15.2)	52 (23.2)	0.061^b^
Use of antidepressants before sepsis^c^, no. of patients (%)	8 (5.4)	4 (15.4)	1 (2.3)	13 (6.0)	55 (24.6)	0.130^b^
Use of analgesics before sepsis^c^, no. of patients (%)	23 (15.1)	8 (29.6)	7 (15.6)	38 (17.0)	55 (24.6)	0.216^b^
*Intensive care measures*						
Length of ICU stay, median (IQR), d	26.0 (30.0)	17.0 (20.0)	28.5 (37.0)	25.0 (31.0)	18 (8.0)	0.155^a^
Mechanical ventilation, no. of patients (%)	128 (84.8)	22 (81.5)	39 (86.7)	189 (84.8)	1 (0.4)	0.839^b^
Mechanical ventilation, number of days, if applicable, mean (SD)	14.7 (16.2)	7.6 (12.2)	14.5 (19.0)	13.7 (16.3)	102 (45.5)	0.649^a^
Renal replacement therapy, no. of patients (%)	39 (26.0)	7 (25.9)	14 (31.1)	60 (27.0)	2 (0.9)	0.788^b^
Use of sedatives, no. of patients (%)	80 (79.2)	10 (55.6)	19 (73.1)	109 (75.2)	79 (35.3)	0.098^b^
Psychiatric diagnosis at ICU discharge, no. of patients, (%)	28 (19.3)	7 (26.9)	6 (13.6)	50 (18.0)	9 (4.0)	0.435^b^

Trajectory classes: mr, mild recovered trajectory; sp, severe persistent trajectory; sr, severe recovered trajectory

IQR, interquartile range; ICU, intensive care unit; SD, standard deviation

^a^Kruskal–Wallis test

^b^Chi-square test

^c^Documented by primary care records 3 months before ICU

One month after ICU discharge, patients with mild depressive symptoms reported statistically significantly better health-related quality of life than patients with moderate to severe depressive symptoms, as indicated by all subscales of the Short Form Health 36 (SF-36) (Kruskal–Wallis test, *p* < 0.05, for each subscale). Details are shown in Table 2.

**Table 2:** Health-related quality of life (HRQOL) 1 month after discharge from ICU allocated to trajectory classes

	Mild recovered (*n* = 152)	Severe persistent (*n* = 27)	Severe recovery (*n* = 45)	Total (*n* = 224)	Missings no. (%)	*p* value		
						mr versus sp	mr versus sr	sp versus sr
*Quality of life (SF-36)*								
Physical functioning (PF), mean (SD)	16.9 (24.6)	8.1 (20.4)	6.1 (19.7)	13.5 (23.7)	9 (4.0)	** < 0.001**^**a**^	** < .035**^**a**^	.456^a^
Role physical (RP), mean (SD)	11.7 (26.1)	1.0 (4.9)	2.8 (12.3)	9.0 (23.1)	0 (0.0)	** < 0.016**^**a**^	** < .012**^**a**^	.737^a^
Bodily pain (BP), mean (SD)	60.3 (35.5)	34.7 (34.4)	37.1 (34.1)	52.8 (36.9)	5 (2.2)	** < 0.002**^**a**^	** < .001**^**a**^	.924^a^
General health (GH), mean (SD)	44.6 (18.8)	34.9 (17.6)	36.2 (17.0)	41.4 (18.4)	3 (1.3)	** < 0.005**^**a**^	** < .007**^**a**^	.584^a^
Vitality (VT), mean (SD)	39.4 (20.1)	24.6 (15.4)	25.4 (17.8)	35.0 (18.4)	0 (0.0)	** < 0.001**^**a**^	** < .001**^**a**^	.943^a^
Social functioning (SF), mean (SD)	79.4 (28.7)	62.0 (37.0)	66.6 (35.5)	71.1 (31.6)	3 (1.3)	** < 0.015**^**a**^	** < .033**^**a**^	.555^a^
Role emotional (RE), mean (SD)	61.9 (46.6)	33.3 (47.1)	31.8 (41.9)	51.2 (47.5)	2 (0.9)	** < 0.004**^**a**^	** < .001**^**a**^	.949^a^
Mental health (MH), mean (SD)	67.5 (18.5)	46.5 (25.2)	42.1 (17.1)	60.2 (22.2)	0 (0.0)	** < 0.001**^**a**^	** < .001**^**a**^	.390^a^

Bold numbers mean: *p* value of 0.05 or less

HRQOL at baseline is shown by the eight subdomains of the Short Form-36 Questionnaire (SF-36). Range of possible scores: 0–100. High scores indicate low impairment

Trajectory classes: mr, mild recovered trajectory; sp, severe persistent trajectory; sr, severe recovered trajectory

ICU, intensive care unit; SD, standard deviation

^a^Kruskal–Wallis test

Chronic pain intensity and disability from chronic pain measured with Graded Chronic Pain Scale (GCPS) differed significantly between the three trajectory classes at all follow-up assessments: Patients with the mild recovered depressive symptom trajectory had significantly lower GCPS scores than patients with severe persistent or severe recovered trajectory at each follow-up as displayed in Table 3 (Kruskal–Wallis test, *p* < 0.05). Patients with severe persistent depressive symptoms showed the highest GCPS scores. GCPS scores decreased over 1 year parallel to depressive symptoms in patients with the severe recovered trajectory.

**Table 3:** Clinical courses of depressive symptoms, symptoms of posttraumatic stress disorder (PTSD) and chronic pain allocated to trajectory classes

	Mild recovered (*n* = 152)	Severe persistent (*n* = 27)	Severe recovery (*n* = 45)	Total (*n* = 224)	Missings no. (%)	*p* value		
						mr versus sp	mr versus sr	sp versus sr
*Depression*								
MDI 1 month after ICU discharge, median (IQR)	14.0 (10.0)	23.0 (15.0)	29.0 (7.0)	17.0 (15.0)		** < 0.001**^**a**^	** < 0.001**^**a**^	** < 0.025**^**a**^
MDI 6 months after ICU discharge, median (IQR)	7.0 (8.0)	21.0 (19.0)	11.0 (9.0)	8.0 (10.0)		** < 0.001**^**a**^	** < 0.001**^**a**^	** < 0.001**^**a**^
MDI 12 months after ICU discharge, median (IQR)	6.0 (8.0)	26.0 (12.0)	6.8 (10.0)	7.0 (10.0)		** < 0.001**^**a**^	0.172^a^	** < .001**^**a**^
*Chronic pain (GCPS)*								
Pain intensity 1 month after ICU discharge, median (IQR)	43.3 (36.7)	60.0 (23.3)	53.3 (7)	46.7 (33.3)	33 (14.7)	** < 0.001**^**a**^	** < .010**^**a**^	0.199^**a**^
Pain intensity 6 months after ICU discharge, median (IQR)	33.3 (36.7)	53.3 (28.3)	40.0 (30.0)	36.7 (33.3)	33 (14.7)	** < 0.001**^**a**^	0.229^**a**^	** < 0.007**^**a**^
Pain intensity 12 months after ICU discharge, median (IQR)	30.0 (38.3)	41.7 (38.3)	40.0 (40.0)	33.3 (36.7)	33 (14.7)	** < 0.009**^**a**^	** < .038**^**a**^	0.413^**a**^
Disability from pain 1 month after ICU discharge, median (IQR)	23.3 (50.0)	56.7 (60.0)	50.0 (66.7)	33.3 (63.3)	33 (14.7)	** < 0.001**^**a**^	** < 0.001**^**a**^	0.548^**a**^
Disability from pain 6 months after ICU discharge, median (IQR)	20.0 (40.0)	56.7 (70.0)	30.0 (50.0)	26.7 (50.0)	33 (14.7)	** < 0.001**^**a**^	0.230^**a**^	** < 0.005**^**a**^
Disability from pain 12 months after ICU discharge, median (IQR)	13.3 (36.7)	40.0 (50.0)	20.0 (50.0)	20.0 (46.7)	33 (14.7)	** < 0.001**^**a**^	0.130^**a**^	** < 0.042**^**a**^
*Posttraumatic stress*								
PTSS-10 score 1 month after ICU discharge, median (IQR)	17.0 (10.0)	34.5 (9.0)	30.5 (15.0)	22.0 (15.0)	1 (0.4)	** < 0.001**^**b**^	** < 0.001**^**b**^	0.367^b^
PTSS-10 score 6 months after ICU discharge, median (IQR)	17.0 (9.0)	40.0 (16.0)	23.5 (16.0)	19.0 (13.0)	18 (8.0)	** < 0.001**^**b**^	** < 0.001**^**b**^	** < 0.001**^**b**^
PTSS-10 score 12 months after ICU discharge, median (IQR)	17.0 (10.0)	41.0 (19.0)	20.0 (13.0)	20.0 (14.0)	22 (9.8)	** < 0.001**^**b**^	0.069^b^	** < 0.001**^**b**^
PTSS-10 score > 34 1 month after ICU discharge, no. (%)	7.0 (4.6)	13.0 (48.1)	15.0 (33.3)	35.0 (15.7)	1 (0.4)	** < 0.001**^**c**^	** < 0.001**^**c**^	0.212^c^
PTSS-10 score > 34 6 months after ICU discharge, no. (%)	6.0 (4.3)	14.0 (53.8)	7.0 (17.1)	27.0 (13.1)	18 (8.0)	** < 0.001**^**c**^	** < 0.006**^**c**^	** < 0.002**^**c**^
PTSS-10 score > 34 12 months after ICU discharge, no. (%)	8.0 (5.8)	15.0 (62.5)	5.0 (12.2)	28.0 (13.9)	22 (9.8)	** < 0.001**^**c**^	0.170^c^	** < 0.001**^**c**^

Bold numbers mean: *p* value of 0.05 or less

Sum scores of Major Depression Inventory (MDI), for depression, Graded Chronic Pain Scale (GCPS), for pain intensity and resulting disability, and Posttraumatic Stress Scale (PTSS-10), for symptoms of PTSD. High scores indicate high impairment. Range of possible sum scores: MDI: 0–50, GCPS intensity/disability: 0–100, PTSS-10: 10–70. PTSS-10 scores over 34 are considered to confirm the diagnosis of PTSD

Trajectory classes: mr, mild recovered trajectory; sp, severe persistent trajectory; sr, severe recovered trajectory

MDI, major depression inventory; GCPS, Graded Chronic Pain Scale; PTSS-10, Posttraumatic Stress Scale; ICU, intensive care unit; IQR, interquartile range

^a^Kruskal–Wallis test

^b^Mann–Whitney test

^c^Chi-square test

Similar results were found for symptoms of posttraumatic stress. Patients with the mild recovered trajectory had the lowest PTSS-10 scores, patients with the severe persistent trajectory had the highest PTSS-10 scores, and patients that recovered from severe depressive symptoms had also decreasing PTSS-10 scores over 1 year as displayed in Table 2 (Kruskal–Wallis test, *p* < 0.05). In a linear regression model of multiple covariates, no sociodemographic or clinical parameter was associated with the course of PTSD or pain.

### Further analysis

Assuming an association between PTSD and chronic pain with the trajectory of depressive symptoms, we performed a multinomial regression. This showed that an increase in PTSS-10 score was associated with a severe persistent or severe recovered trajectory (instead of a mild recovered trajectory) with odds ratio (OR) 1.167 (95% CI [1.105–1.232], *p* < 0.001) and OR 1.157 (95% CI [1.103–1.213], *p* < 0.001), respectively. GCPS score trajectories showed similar associations (ORs of 1.016 (95% CI [1.002–1.030], *p* < 0.03) and 1.012 (95% CI [1.001–1.024], *p* < 0.04) with the severe persistent and severe recovered trajectory, respectively.

**Fig. 3:**
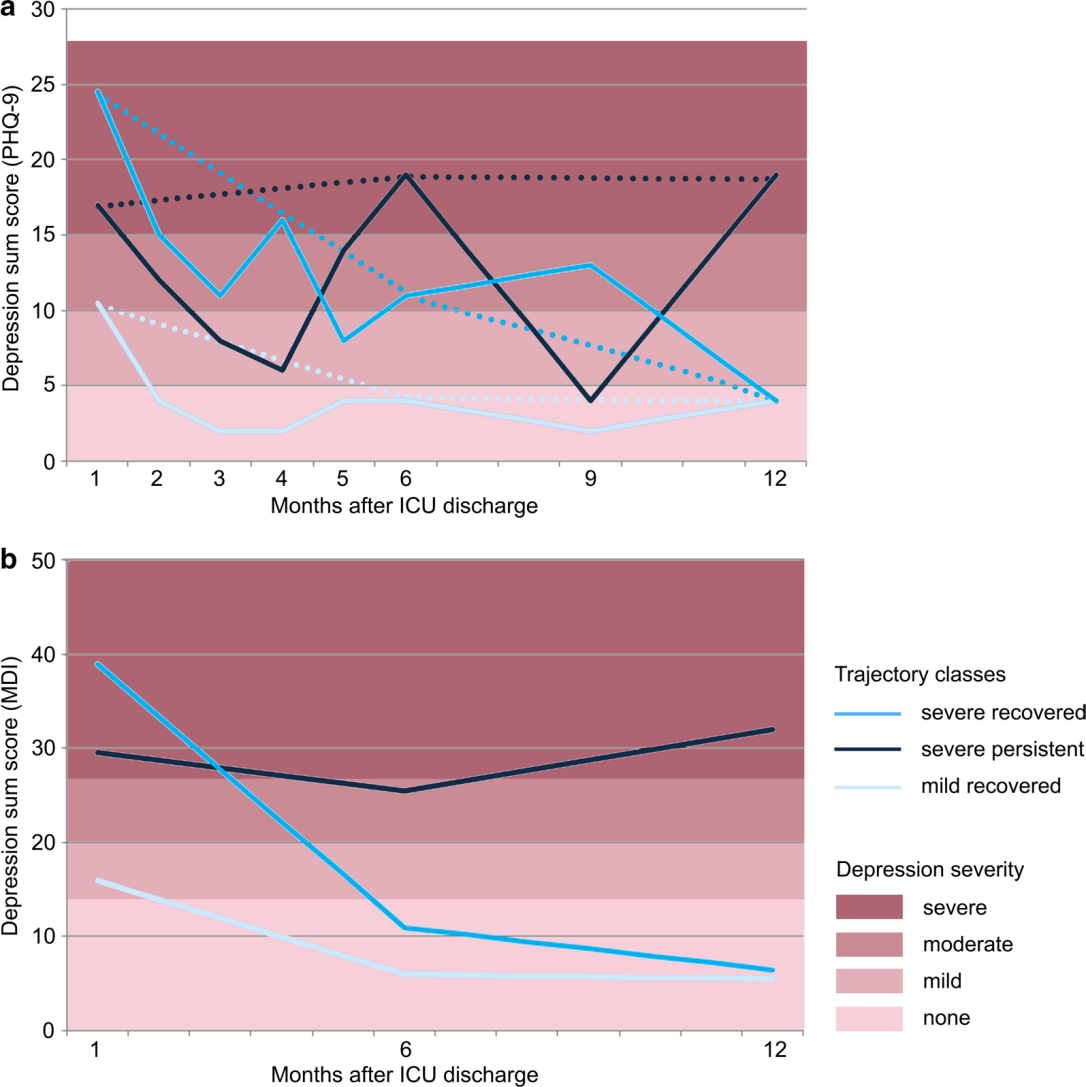
Trajectories of depressive symptoms within the intervention group: Trajectories of depressive symptoms are shown by median Major Depression Inventory (MDI) and Patient Health Questionnaire 9 (PHQ-9) sum scores of the trajectory classes. High scores indicate high impairment. Range of possible sum scores: MDI 0–50, PHQ-9: 0–27. Diagnostic thresholds for depression are labeled according to ICD-10 and DSM-IV. **a** PHQ-9 scores at 1, 2, 3, 4, 5, 6, 9 and 12 months after ICU discharge (high-frequency follow-up). The dashed lines show the trajectories considering only the 1-, 6- and 12-month follow-up. **b** MDI at 1, 6 and 12 months after ICU discharge (low-frequency follow-up)

The subgroup analysis for patients of the intervention group with at least two available MDI scores and four available PHQ-9 scores identified similar trajectories of the three trajectory classes (mild recovered, severe persistent, severe recovered). At this, high-frequency follow-ups of the PHQ-9 scores showed high fluctuations, which could not be detected by lower follow-up frequencies (see Fig. 3).

## Discussion

The aim of this post hoc analysis was to explore trajectories of depressive symptoms in sepsis survivors. Three classes of trajectories could be identified, with mild recovered, severe persistent and severe recovered levels of depressive symptoms. These three trajectories could be demonstrated by analyzing two different depression scales.

Trajectories of depressive symptoms have already been described in the literature: Petersen and colleagues identified two latent trajectory classes of symptoms (fast improvers with declining symptoms and slow improvers with persistent symptoms) in their post hoc analysis of a RCT on a primary care-based case management intervention for patients with major depression [33].

A large Australian cohort study analyzed depression trajectories 24 months following traumatic brain injury, also using Growth Mixture Modeling. Three groups with low, high and delayed depression were identified [34]. Authors concluded that it is necessary to take considerable fluctuations in depression trajectories into account. A similar US study even categorized four groups, with low depression (70.1%), delayed depression (13.2%), depression recovery (10.4%) and persistent depression (6.3%) 12 months after traumatic brain injury [35].

Within our analysis, patients with moderate to severe depressive symptoms at ICU discharge showed reduced mental and functional health-related quality of life (HRQOL). Thus, identification of risk factors for depression at ICU discharge seems crucial. In particular, patients of the severe persistent trajectory class would benefit from a particular focus.

Looking for trajectory predictors at ICU discharge, we found an association between depression severity and chronic pain: Chronic pain is frequently reported by septic and non-septic critical care survivors, even if there was no preexisting pain condition [36]. A recent systematic review [37] concluded that comorbid pain and depression interact with each other resulting in increased outcome severity. Membership of the severe persistent or the severe recovered trajectory class was associated with increasing PTSD symptom severity. This goes along with a large prospective UK cohort study [7]: Nearly five thousand patients after critical illness were evaluated with established questionnaires for depression, anxiety and PTSD. The presence of one psychiatric disorder was associated with a 65% chance of co-occurrence with symptoms of one of the other two disorders.

No other significant factors associated with one of the trajectory classes were found. In this respect, reported evidence in literature seems inconsistent: A large Scandinavian study of nearly six thousand patients with traumatic injuries found female gender, age and hospital length of stay as independent predictors of depressive symptoms after hospital discharge among others [38]. The Australian cohort study found an association between depression and shorter periods of hospitalization [34]. A recent systematic review and meta-analysis demonstrated prior comorbid psychopathology, but neither age, sex or severity of illness nor ICU or hospital length of stay, use of benzodiazepines or duration of sedation being associated with depressive symptoms in critical illness survivors [12].

In summary, our results support timely screening for actual psychiatric symptoms and pain following discharge from ICU. Furthermore, also later occurrence of posttraumatic symptoms or pain should draw attention to the course of depressive symptoms, as symptom aggravation seems likely [9].

Even if statistically not significant, patients with the severe persistent trajectory showed lower education and higher rates of unemployment, prior psychiatric history and use of antidepressants/analgesics before ICU. These factors highlight the importance of a largely unexplored area in need of investigation—namely, the identification and potential contribution of pre-morbid traits to the development of depression post-ICU.

Results from high-frequency follow-up data analysis suggest high fluctuations in the course of depression. Due to the restricted sample size, depression trajectories may have been affected by strong fluctuations of individual patients. These fluctuations could be missed in low-frequency follow-up.

Our study has several strengths and limitations. It includes—to our knowledge—the first trajectory analysis of depressive symptoms in sepsis survivors. Follow-up frequency and number of applied scales were high, and the size of our prospectively observed cohort was large.

As this is a retrospective post hoc analysis, results are only exploratory. Data were mostly obtained in telephone interviews, and conclusion therefore may differ from face-to-face interviews. However, telephone assessment of mental health with standardized questionnaires has been established in primary care research [39]. Patients were distributed unequally to the three trajectory classes, and the number of patients in two classes was relatively small. Due to the high mortality in our population, there is a possible risk of selection bias, as 41 patients (14%) died before 6-month follow-up. Moreover, 26 patients (12%) dropped out before 6-month follow-up due to other reasons. These numbers correspond to the primary analysis and have been discussed in the parent paper [16].

Differences in therapy after ICU discharge and existing conditions pre-ICU were not fully recorded and can therefore not be excluded as a confounder. However, no major effect of the SMOOTH trial intervention on depression trajectories was assumed, as there no influence on overall depression severity had been detected. Lack of intervention specificity is considered to be the main reason; for a detailed discussion, we refer to the parent paper [16].

Results may be skewed by physical impairments like sleeplessness which might have affected the sum scores. Furthermore, the content overlap between several items of the used measures in depression, HRQOL and pain needs to be taken into account. Thus, the questionnaires we used do not substitute a clinical interview by a psychiatrist to confirm the diagnosis of depression. However, both measures are considered for daily clinical work with restricted resources [40].

## Conclusions

Three different trajectories of depressive symptoms over 1 year after discharge from ICU could be identified in sepsis survivors, with mild recovered, severe persistent and severe recovered levels of depressive symptoms. More than one-third of the observed patients did not recover from depression. Course and severity of depression appear to be associated with chronic pain, PTSD and reduced HRQOL. No other associated factors of the trajectories were found. Regular screening of sepsis survivors on symptoms of depressions, chronic pain and PTSD within 1 year after ICU seems advisable. The role of pre-existing characteristics in the development of distinct patterns of depressive symptoms should be considered.

## Data Availability

The datasets used and/or analyzed during the current study are available from the corresponding author on reasonable request.

## Abbreviations

BIC: Bayesian information criterion
GCPS: Graded Chronic Pain Scale
GMM: Growth Mixture Modeling
HRQOL: Health-related quality of life
ICU: Intensive care unit
IQR: Interquartile range
MDI: Major Depression Inventory
OR: Odds ratio
PICS: Post-intensive care syndrome
PCP: Primary care physician
PHQ-9: Patient Health Questionnaire 9
PTSD: Posttraumatic stress disorder
PTSS-10: Posttraumatic Stress Scale
RCT: Randomized controlled trial
SF-36: Short Form-36 Questionnaire
SMOOTH: Sepsis survivors Monitoring and Coordination in Outpatient Healthcare

## References

[1] SepNet Critical Care Trials Group (2016). Incidence of severe sepsis and septic shock in German intensive care units: the prospective, multicentre INSEP study. Intensive Care Med.

[2] Vincent J-L, Sakr Y, Sprung CL (2006). Sepsis in European intensive care units: results of the SOAP study. Crit Care Med.

[3] Needham DM, Davidson J, Cohen H (2012). Improving long-term outcomes after discharge from intensive care unit: report from a stakeholders' conference. Crit Care Med.

[4] Davydow DS, Gifford JM, Desai SV (2009). Depression in general intensive care unit survivors: a systematic review. Intensive Care Med.

[5] Winters BD, Eberlein M, Leung J (2010). Long-term mortality and quality of life in sepsis: a systematic review. Crit Care Med.

[6] Desai SV, Law TJ, Needham DM (2011). Long-term complications of critical care. Crit Care Med.

[7] Hatch R, Young D, Barber V (2018). Anxiety, depression and post traumatic stress disorder after critical illness: a UK-wide prospective cohort study. Crit Care.

[8] Gros DF, Price M, Magruder KM (2012). Symptom overlap in posttraumatic stress disorder and major depression. Psychiatry Res.

[9] Ravn SL, Hartvigsen J, Hansen M (2018). Do post-traumatic pain and post-traumatic stress symptomatology mutually maintain each other? A systematic review of cross-lagged studies. Pain.

[10] Outcalt SD, Kroenke K, Krebs EE (2015). Chronic pain and comorbid mental health conditions: independent associations of posttraumatic stress disorder and depression with pain, disability, and quality of life. J Behav Med.

[11] Bair MJ, Robinson RL, Katon W (2003). Depression and pain comorbidity: a literature review. Arch Intern Med.

[12] Kurdyak P, Cairney J (2011). Predicting recurrence of depression. CMAJ.

[13] Rabiee A, Nikayin S, Hashem MD (2016). Depressive symptoms after critical illness: a systematic review and meta-analysis. Crit Care Med.

[14] Nübel J, Guhn A, Müllender S (2020). Persistent depressive disorder across the adult lifespan: results from clinical and population-based surveys in Germany. BMC Psychiatry.

[15] Jensen JF, Thomsen T, Overgaard D (2015). Impact of follow-up consultations for ICU survivors on post-ICU syndrome: a systematic review and meta-analysis. Intensive Care Med.

[16] Schmidt K, Worrack S, von Korff M (2016). Effect of a primary care management intervention on mental health-related quality of life among survivors of sepsis: a randomized clinical trial. JAMA.

[17] Schmidt K, Thiel P, Mueller F (2014). Sepsis survivors monitoring and coordination in outpatient health care (SMOOTH): study protocol for a randomized controlled trial. Trials.

[18] Schmidt KF, Schwarzkopf D, Baldwin L-M (2020). Long-term courses of sepsis survivors: effects of a primary care management intervention. Am J Med.

[19] Bech P, Rasmussen N-A, Olsen L (2001). The sensitivity and specificity of the Major Depression Inventory, using the Present State Examination as the index of diagnostic validity. J Affect Disord.

[20] Kroenke K, Spitzer RL, Williams JB (2001). The PHQ-9: validity of a brief depression severity measure. J Gen Intern Med.

[21] Hahn D, Reuter K, Härter M (2006). Screening for affective and anxiety disorders in medical patients-comparison of HADS, GHQ-12 and Brief-PHQ. Psychosoc Med.

[22] Lins L, Carvalho FM (2016). SF-36 total score as a single measure of health-related quality of life: scoping review. SAGE Open Med.

[23] Stoll C, Kapfhammer HP, Rothenhäusler HB (1999). Sensitivity and specificity of a screening test to document traumatic experiences and to diagnose post-traumatic stress disorder in ARDS patients after intensive care treatment. Intensive Care Med.

[24] Elliott AM, Smith BH, Smith CW (2000). Changes in chronic pain severity over time: the Chronic Pain Grade as a valid measure. Pain.

[25] Proust-Lima C, Philipps V, Liquet B (2017). Estimation of extended mixed models using latent classes and latent processes: the R package lcmm. J Stat Softw.

[26] R Core Team. R: a language and environment for statistical computing. https://www.R-project.org/.

[27] Ram N, Grimm KJ (2009). Growth mixture modeling: a method for identifying differences in longitudinal change among unobserved groups. Int J Behav Dev.

[28] Schwarz G (1978). Estimating the dimension of a model. Ann Stat.

[29] Wickham H (2016). ggplot2: Elegant graphics for data analysis: use R!.

[30] Microsoft Corporation. Microsoft Excel. https://office.microsoft.com/excel.

[31] Kruskal WH, Wallis WA (1952). Use of ranks in one-criterion variance analysis. J Am Stat Assoc.

[32] IBM Corporation. IBM SPSS STatistics for Windows: version 26.0. https://hadoop.apache.org.

[33] Petersen JJ, Hartig J, Paulitsch MA (2018). Classes of depression symptom trajectories in patients with major depression receiving a collaborative care intervention. PLoS ONE.

[34] Gomez R, Skilbeck C, Thomas M (2017). Growth mixture modeling of depression symptoms following traumatic brain injury. Front Psychol.

[35] Bombardier CH, Hoekstra T, Dikmen S (2016). Depression trajectories during the first year after traumatic brain injury. J Neurotrauma.

[36] Baumbach P, Götz T, Günther A (2016). Prevalence and characteristics of chronic intensive care-related pain: the role of severe sepsis and septic shock. Crit Care Med.

[37] IsHak WW, Wen RY, Naghdechi L (2018). Pain and depression: a systematic review. Harv Rev Psychiatry.

[38] Ahl R, Lindgren R, Cao Y (2017). Risk factors for depression following traumatic injury: an epidemiological study from a scandinavian trauma center. Injury.

[39] Evans M, Kessler D, Lewis G (2004). Assessing mental health in primary care research using standardized scales: can it be carried out over the telephone?. Psychol Med.

[40] Lowe B (2004). Comparative validity of three screening questionnaires for DSM-IV depressive disorders and physicians diagnoses. J Affect Disord.

